# Household Food Insecurity Is Associated with Parental Perceptions of and Student Participation in School Meals

**DOI:** 10.3390/nu16193375

**Published:** 2024-10-04

**Authors:** Monica D. Zuercher, Juliana F. W. Cohen, Christina A. Hecht, Kenneth Hecht, Dania Orta-Aleman, Deborah A. Olarte, Leah E. Chapman, Margaret Read, Lorrene D. Ritchie, Wendi Gosliner

**Affiliations:** 1 Nutrition Policy Institute, Division of Agriculture and Natural Resources, University of California, 1111 Franklin Street, 11th Floor, Oakland, CA 94607, USA; ceahecht@ucanr.edu (C.A.H.); kenhecht@ucanr.edu (K.H.); dorta@ucanr.edu (D.O.-A.); lritchie@ucanr.edu (L.D.R.); wgosliner@ucanr.edu (W.G.); 2 Center for Health Inclusion, Research and Practice (CHIRP), Merrimack College, 315 Turnpike Street, North Andover, MA 01845, USA; jcohen@hsph.harvard.edu (J.F.W.C.);; 3 Harvard T.H. Chan School of Public Health, 677 Huntington Ave, Boston, MA 02115, USA; 4 Department of Nutrition and Food Studies, Steinhardt School of Culture, Education, and Human Development, New York University, 411 Lafayette Street, 5th Floor, New York, NY 10003, USA; ddo223@nyu.edu; 5 Partnership for a Healthier America, Prince Frederick, MD 20678, USA; mread@ahealthieramerica.org

**Keywords:** NSLP, SBP, food insecurity, universal school meals, parental perceptions

## Abstract

Background/Objectives: School meals are an important source of nutrition for children and have been found to help mitigate food insecurity. This study evaluated the association between food insecurity and school meal participation and whether parental perceptions about school meals differ by food security status. Methods: In May 2022, 1110 Californian parents of K-12 students shared their perceptions about school meals, including meal quality, healthiness, stigma, and benefits, as well as their child’s participation in school meals, in an online survey. Household food security was determined using the USDA 6-item module. Logistic and Poisson regression models were used for analysis. Results: The prevalence of household food insecurity was 56.2% (69.6% in households of students eligible for free meals, 55.9% in reduced-price, and 38.3% in non-eligible). Many of the reported benefits of school meals (saving families money and time) were equally highly endorsed by parents with and without food insecurity (*p* > 0.05). Parents reporting food insecurity had less favorable perceptions of school meals and perceived more stigma (*p* < 0.05). Food insecurity was positively associated with breakfast participation, especially among elementary school students and students not eligible for free or reduced-price meals (FRPMs) (*p* < 0.05). Conclusions: Food insecurity is prevalent among California families with school-age children, even in families not eligible for federal FRPMs. Food-insecure households have more negative perceptions of school meals and experience more stigma, though they also report higher breakfast participation. Improving school meal quality and appeal, ensuring parents are familiar with meal quality and healthfulness, and reducing stigma may ease food insecurity while improving children’s health.

## 1. Introduction

Household food insecurity refers to limited access to adequate food for active and healthy living due to lack of money and other resources [1]. Household food insecurity can vary from “low food security”, which is characterized by a reduced quality, variety, or desirability of the diet with little or no reduced food intake, to “very low food security”, which includes indications of disrupted eating patterns and reduced food intake [2]. In the U.S., 10.2% of households experienced food insecurity in 2021 (6.4% experienced low food security and 3.8% experienced very low food security), and this prevalence was higher among households with children (12.5%) [1]. Food insecurity has been associated with negative outcomes in children and adolescents, including inadequate intake of essential nutrients, increased risk for chronic diseases, poor academic performance, behavioral and emotional issues, and socio-familial disturbances [3,4].

Healthy foods, which have higher nutrient density, have been associated with a higher cost per kilocalorie, whereas unhealthy foods, with higher energy density and lower nutrient density, have been associated with lower cost [5,6]. As a result, children in food-insecure households generally have limited access to high-quality foods [3,5,6,7]. Moreover, low-income neighborhoods often have poorer food environments, with fewer full-service grocery stores and greater availability of fast-food restaurants, and these neighborhoods’ food retailers sell disproportionately more energy-dense, nutrient-poor food [7]. In California, economically disadvantaged neighborhoods were found to sell poorer quality and more expensive fruits and vegetables [8]. As a consequence, food insecurity has been associated with lower dietary quality, lower consumption of fruits, vegetables, and dairy, and higher consumption of fast food and sugar-sweetened beverages in children and adolescents [4,9].

The National School Lunch Program (NSLP) and the School Breakfast Program (SBP) are federal meal programs that provide nutritionally balanced meals at low- to no-cost to children attending public and non-profit private schools each school day and have been found to help mitigate food insecurity [1,3,10,11,12]. School meals are an important source of nutrition for children, contributing a higher proportion of daily energy intake for children from households with low income than those with higher income [13]. Participation in school meal programs has been associated with multiple other benefits for students with food insecurity and/or low-income students, including improvements in dietary intake, reading scores, and reductions in the probability of being overweight [1,10,14,15,16,17]. However, when school meals are not available (e.g., during the summer and winter breaks or emergency school closures), food insecurity increases and diet quality declines [6,17,18,19].

Traditionally, children were eligible to receive free or reduced-price school meals (FRPMs) based on their household income level, participation in certain federal assistance programs, or status as a homeless, migrant, runaway, or foster child [11]. Alternatively, they could attend a school that participated in one of the different provisions the USDA has available to allow high-poverty schools to serve school meals at no cost to all students [11,20]. Children not qualifying for FRPMs could purchase a “full price” school meal (still subsidized by the federal government, with the cost varying by school districts across the U.S.). However, this tiered system has been criticized by advocates for multiple reasons. For example, this classification based on household income and size ignores factors like cost of living, which is strongly associated with food insecurity [4]. Moreover, income-based access to school meals may create stigma related to meal participation and reductions in student participation, as students worry about being perceived as being from a household with low income [21]. Universal school meal (USM) programs—where all students have access to school meals at no charge—can increase student participation by changing the perception of school meals being primarily for children from families with low income to a more inclusive experience of school meals being a healthy source of food for all students [21,22]. In addition to the reduction in stigma and increase in student participation in school meals, USM has been associated with improvements in diet quality, school attendance, academic performance, food security, and household income [14,16,22,23,24].

Little is known about how food security status affects the association between parental perceptions and student participation in school meals, especially in the context of statewide USM programs. While previous research indicates that student participation in school meals varies by school level and FRPM eligibility, there has been limited investigation into whether these factors influence the relationship between food insecurity and student participation in school meals [4,25,26]. Thus, the first aim of this study was to evaluate whether parental perceptions about school meals differ by food security status. The second aim was to assess the relationship between food insecurity and student participation in school meals and to determine if this relationship varies based on school level or FRPM eligibility.

## 2. Materials and Methods

### 2.1. Study Design and Participants

The participants of this cross-sectional study included parents, guardians, and other caregivers with one or more children attending a public or charter elementary, middle, or high school in California [grades kindergarten through 12 (K-12)]. Parents of students who attended a private school or home school, who attended school exclusively remote during the 2021–2022 school year, or who do not live in California were excluded.

Our study aimed to recruit 1000 parents of students who reflect the characteristics of California school students with regard to race/ethnicity, FRPM eligibility, and state region [27,28,29]. Details on the sampling quotas for each category have been described elsewhere [30].

### 2.2. Recruitment

Parents in a private research panel (*n* = 152,000) were invited via email and text to complete an online survey sharing their perspectives about school meals during the school year 2021–2022 [31]. The invitation was sent in May 2022, and the survey link remained open for three weeks until the survey quotas were reached. Parents who clicked on the survey link but did not meet the eligibility criteria or fell into a closed quota category (*n* = 2012) were thanked and did not complete the survey. The final sample consisted of 1110 participants.

### 2.3. Survey Instrument

The survey was developed by the research team in collaboration with external experts in research, policy, and community-based programs and included validated items when available [32,33,34]. Posteriorly, it was pilot-tested by a small group of parents of K-12 students from diverse races, ethnicities, and socioeconomic backgrounds. The final survey included 10 screener questions and 34 questions assessing different aspects of the school meal programs, taking participants approximately 20 min to complete (Appendix A). Parents with more than one child in grades K-12 were asked to focus their responses on their child with the most recent birthday.

The survey was programmed in Qualtrics to be accessed online with a phone, computer, or tablet, and it was available in English or Spanish (Qualtrics Version March 2022, Provo, UT, USA). Parents received a USD 20 thank-you gift card for completing the survey.

### 2.4. Measures

#### 2.4.1. Parental Perceptions

The survey included multiple questions about their perceptions of school lunch and breakfast (only for parents who reported that their child’s school currently offers breakfast), as well as questions about school meals generally. Response options utilized a 5-point Likert scale, but for analysis purposes, response options were dichotomized as 1: agree and strongly agree; 0: strongly disagree, disagree, and neither agree nor disagree. “Don’t know” responses were excluded.

#### 2.4.2. Student Participation

Frequency of lunch participation was obtained with the question, “In a typical week this school year, how often do you think your child eats a school lunch (lunch served by the school and NOT brought from home)?” with the answer options ranging from “No days” to “5 days”. Breakfast participation was measured using a similar question.

#### 2.4.3. Food Insecurity

Household food security was assessed using a six-item scale [35]. This scale categorizes households into high or marginal food security, low food security, or very low food security based on the number of affirmative responses to six statements about their food situation over the past 12 months [35]. A parent was classified as experiencing household food insecurity if their food security level was low or very low and as having household food security if their level was high or marginal.

#### 2.4.4. Covariates

Covariates were chosen based on the literature showing that these factors may be related to food insecurity, student participation in school meals, and/or parental perceptions of school meals [9,15,25,26,30]. The covariates used in analyses included student race/ethnicity, school urbanicity, and the total number of children under 18 years old living with the parent. The parent reported the student’s race and ethnicity, and for analysis purposes, response options were combined into five categories: Non-Hispanic white, Hispanic, Black/African American, Asian/Asian American, and Other race/Multiracial (due to small sample sizes, this category included Alaska Native/American Indian and Native Hawaiian/Other Pacific Islander). Urbanicity was determined using the zip code of the school reported by each parent and classified as urban (RUCA primary code = 1) or non-urban (RUCA primary codes = 2–10) [36]. FRPM eligibility of the student was classified as eligible for free meals [(family income < 130% of the federal poverty line (FPL)], eligible for reduced-price meals (family income between 130 and 185% of the FPL), and non-eligible (family income > 185% of FPL), based on the parent’s self-reported household size and income [37].

### 2.5. Statistical Analysis

Frequencies and percentages were used to describe categorical variables. Chi-squared tests were used to compare parental perceptions by food security level. Logistic regression models were used to evaluate if each parent’s perception differs by food security level while adjusting for covariates. Poisson regression models were used to evaluate the association between food security level and frequency of school meal participation while adjusting for covariates. The interaction of food insecurity with school level and FRPM eligibility was evaluated by adding interaction terms in separate models. Stratified models were fitted if needed. All of the statistical analyses were conducted in Stata using a significance level of α = 0.05 and α = 0.20 for interaction terms.

## 3. Results

### 3.1. Sample Characteristics

Over half of the parents who completed the survey experienced household food insecurity (56.2%). Most respondents were the mothers of the students (85.9%) and used English as their preferred language (80.5%) (Table 1). Nearly half of parents had children who were eligible for free meals (43.6%). Most respondents were the parents of students in elementary school (58.6%), and the majority identified as Hispanic ethnicity (51.7%). Compared to parents in households reporting food security, a higher proportion of parents reporting household food insecurity reported being the mother of the student, living in a household of five or more people, having lower income, participating in Supplemental Nutrition Assistance Program (SNAP), having a child who identifies as Hispanic, and having a child who eats school breakfast (*p* < 0.05). Parents in households reporting food security most often reported their child’s race as Asian (*p* < 0.05). There were no statistically significant differences in the preferred language, student’s gender, school level, or lunch participation by food security level (*p* > 0.05).

**Figure 1:**
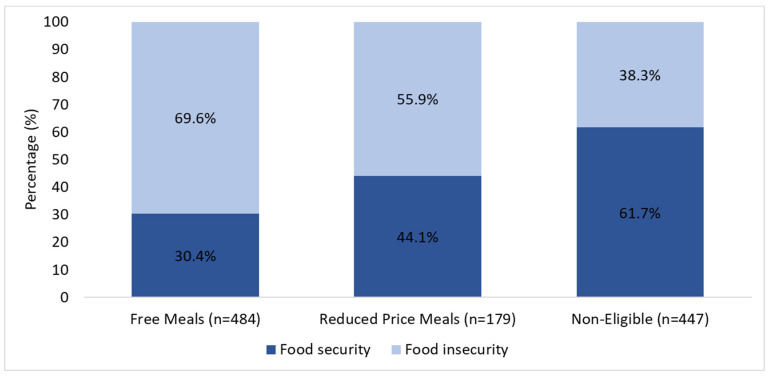
Prevalence of food insecurity among California parents of K-12 students stratified by free and reduced-price meal eligibility. All values are statistically significantly different from each other.

Food insecurity prevalence was highest (69.6%) in households in which the student was eligible for free meals, and while it was lowest in households in which the student was not eligible for free or reduced-price school meals, over a third still reported being food-insecure (38.3%) (Figure 1).

### 3.2. Parental Perceptions about School Breakfast and Lunch by Food Security Level

Compared to parents in households with food security, a lower proportion of parents in households with food insecurity reported that the school breakfasts/lunches (1) are healthy, (2) taste good, and (3) provide enough food for their child to get full. Additionally, parents from food insecure households were less likely to report that (4) the quality of the school breakfast is good (the perception of lunch quality followed the same trend, but the difference was not statistically significant); (5) their child likes the school lunch and (6) has enough time to eat it (the perceptions about liking school breakfast and having enough time to eat it followed the same trend but these differences were not statistically significant) (Table 2) (*p* < 0.05).

Compared to parents in households with food security, a higher proportion of parents in households with food insecurity reported that their child gets tired of the same foods being served at school breakfast and lunch and that they would prefer that their child have the option to eat school breakfast after the bell (*p* < 0.05) (Table 2). There were no statistically significant differences in the percentages of parents who reported that they or their child prefers to eat breakfast at home or bring lunch from home or that they have concerns about the amount of sugar in school breakfasts or lunches (*p* > 0.05).

### 3.3. Parental Perceptions about School Meals by Food Security Level

Compared to parents with food security, a higher proportion of parents in households with food insecurity reported that their child would be embarrassed to eat school meals and that they believe school meals are only for children whose families have low incomes (*p* < 0.05) (Table 3). There were no statistically significant differences by household food security status in the percentage of parents that reported school meals can save their family money, time, or stress, or that think that eating school meals may benefit students academically (*p* > 0.05).

### 3.4. Association between Food Insecurity and Student Participation in School Meals

Being a student in a household with food insecurity (as opposed to food-secure) was positively associated with student participation in school breakfast (*p* = 0.002) but not in school lunch (*p* = 0.35) (Table 4). The interaction terms of food insecurity with school level and FRPM eligibility were statistically significant (*p* for interaction terms < 0.20) (Supplementary Table S1). Stratified analysis by school level showed that the positive association between food insecurity and student participation in school breakfast was only statistically significant among elementary school students (*p* = 0.02).

Stratified analysis by FRPM eligibility showed that food insecurity was positively associated with student participation in school breakfast and school lunch (Table 4) (*p* = 0.01 and *p* = 0.04, respectively). There was no statistically significant association between food insecurity and participation in school meals among students in the FRPM categories (*p* > 0.05).

## 4. Discussion

The prevalence of household food insecurity reported by parents of K-12 students in this California study was higher than the national and state prevalence for families with children. An unexpectedly high proportion (38%) of families not eligible for FRPMs reported being food insecure. Most parents, regardless of their food security status, indicated that school meals help their family save money and time and reduce stress. Parents with food insecurity appeared to have less favorable views on the quality, taste, and healthfulness of school meals and perceived more stigma related to meal participation than parents with food security. Food insecurity was positively associated with student participation in school breakfast, particularly among students in elementary school and those not eligible for FRPMs.

The prevalence of food insecurity among parents of students eligible for free school meals in this study was more than twice the national prevalence among households with incomes below the FPL (69.6% vs. 32.1%, respectively) [1]. Further, almost 40% of parents of children who are not eligible to receive FRPMs reported household food insecurity. A potential explanation for the high prevalence of food insecurity reported in this study could be partly due to the high costs of living in California, which was ranked among the five most expensive states to live in the U.S. in 2022 [38]. In addition to the already high cost of living, inflation, particularly in food prices, during the study period also may have contributed to higher than anticipated levels of food insecurity. These factors are not considered in the eligibility determination for FRPMs in the continental United States [4,39].

Parental perceptions that school meals can help their family save money and time and reduce stress were equally high in parents with and without food insecurity. This finding could partly be explained by the high prevalence of food insecurity among parents of students not eligible for FRPMs. However, it also suggests that parents appreciate the positive benefits of school meals regardless of their food security status. Moreover, families with children face many challenges related to their finances and lack of time or support at all income levels [1,40]. Providing school meals free for all students may free up time and money that families would otherwise have to spend buying and preparing breakfast and lunch for their children on school days.

Parents in households reporting food insecurity have less favorable views on the quality, taste, and healthfulness of school meals than parents in households reporting food security. Multiple factors may play a role in this finding. First, previous studies have reported disparities in the types and healthfulness of foods offered at schools where schools with high socioeconomic levels or predominantly white students were more likely to offer healthier meals and competitive foods than schools with low socioeconomic levels or majority-black or Latino students [41,42,43]. Without data about the healthfulness of the school meals served at the school that the parents are reporting on, it is unknown if the less favorable perceptions are related to a disparity in the quality or variety of school meals offered at their child’s school. Future studies should assess the food provided to determine whether the issue is one of perception or of a differential in the quality of meal programs at schools where more students experience food insecurity. Second, students who are not eligible for FRPMs are more likely to purchase a la carte items; in fact, only 26% of the lunches served through the NSLP in 2019 were served to students who paid full price [44]. Thus, parents of students with food security (and therefore with higher socioeconomic levels and non-eligible for FRPM) may have considered the quality, variety, taste, and healthfulness of a la carte foods rather than those offered through the NSLP. Third, potential “fatigue” in students who eat school meals daily or almost every day may result from the typical 2–4-week cycle menu compared with those students who eat school meals occasionally when there is something they like on the menu. Finally, children in households with food insecurity may be less exposed to healthy and relatively more expensive foods at home, like fruits, vegetables, and dairy, which could make school foods feel less familiar since school meals are required to include fruits, vegetables, whole grain, and lower fat foods [4,9,19,45,46].

Stigma related to school meal participation was higher among parents reporting household food insecurity compared to those reporting household food security. Parents with food insecurity were almost twice as likely to report that their child would be embarrassed to eat school meals than parents with food security. This may be related to the recent history of school meals being associated with poverty, as students eating the school meals may be viewed as disadvantaged by those who bring their own meals or can afford to purchase food [21]. Previous research shows that providing universally free school meals can reduce stigma [14,21,22,23,24]. While data from this study were collected during the pandemic when schools were federally authorized to provide meals to all students at no charge, this temporary provision had only been in place for 2 years, which might not have been enough time to remove perceptions of stigma. Moreover, a handful of states have decided to continue offering universally free school meal programs; therefore, future studies should assess whether stigma related to meal participation remains lower in these states than in states that reverted to the tiered meal eligibility system. Additionally, other policies, such as open campuses (i.e., where students can leave for lunch) or the sales of competitive foods (i.e., snack foods and beverages sold in school), may perpetuate stigma among students who are receiving school meals compared with those who may purchase other options in the presence of universal free school meal policies; these additional factors should also be examined in future studies.

Food insecurity was associated with participation in school breakfast but not school lunch. Historically, student participation in school breakfast has been much lower than participation in school lunch [44,47], as students face barriers specific to breakfast participation, such as arriving at school in time for class and not having enough time to eat breakfast, breakfast being served too early or too late in the morning, and fewer schools offering breakfast. Students from households with food insecurity may be more motivated to overcome these barriers and eat the school breakfast [25,48]. Numerous strategies to increase access to and participation in school breakfast have been suggested, including breakfast after the bell, second chance breakfast, breakfast in the classroom, and grab-and-go options [25,48]. In this study, parents with food insecurity expressed a strong preference for their child’s school to offer breakfast after the bell.

Notably, the association between food insecurity and student participation in school breakfast was only statistically significant among students in elementary school or not eligible for FRPMs. The high participation in school breakfast and lunch among students eligible for FRPMs, regardless of their food security status, could explain the lack of association between food insecurity and student participation among these students. Our results show how, in the context of universal meals, students not eligible for FRPMs who are in households with food insecurity have a higher participation in school breakfast. Moreover, the observed trend of higher levels of stigma among middle and high school students could have contributed to the lack of association between food insecurity and participation in school breakfast among these students (10.0% in elementary school students, 14.3% in middle school students and 14.1% among high school students, *p* = 0.10). The association between food insecurity and student participation highlights the important role that school meals play by increasing access to healthy food for millions of U.S. students, helping them to reduce food insecurity and increase equity. Moreover, these results emphasize how UFSM can help to improve food security for students who would be excluded from receiving school meals free of charge under the traditional eligibility criteria.

Food insecurity and hunger worldwide have worsened in recent years due to various factors, such as the COVID-19 pandemic, war conflicts, weather shocks, and domestic food price inflation [49]. Results from the State of Food Security and Nutrition in the World report indicate that 29.6% of the global population faced food insecurity in 2022, marking an increase of 391 million people compared to 2019 [49]. Ending hunger is part of the 2030 Agenda for Sustainable Development, adopted by all United Nations Member States in 2015 [50]. School meals have been found to directly contribute to sustainable development goals (SDG) 2 zero hunger, SDG4 quality education, and SDG5 gender equality by improving student nutrition, increasing learning capacities and cognitive development, increasing school enrollment and attendance rates, and narrowing gender gaps in access to education and exposure to hunger and malnutrition [51].

This study has several strengths. First, it used a large sample of parents with diverse racial, ethnic, and socioeconomic backgrounds that reflect those of K-12 students in California. Second, it was conducted when schools were authorized to provide school meals to all students at no charge, which provided a unique opportunity to examine the effects of universal school meals on food insecurity and related outcomes. Third, it assessed parental perceptions and student participation in both school lunch and breakfast, which are often studied separately or not at all. Fourth, it used a validated measure of food insecurity widely used in previous studies. Limitations of the study include the fact that the lower boundary of the highest household income category was still slightly low, given California’s living costs. Therefore, we were unable to assess whether truly high-income parents (by California standards) were included in our sample. Another limitation is that we do not know what school students attended and whether the meal quality or other important school meal characteristics differ between students from households with and without food insecurity. Future studies should work to understand this relationship. Additionally, study data were cross-sectional, limiting our ability to establish temporality. Further, data on the frequency of student participation in school meals and some perceptions about how students felt about school meals were based on parents’ self-report of their child’s experiences, which may not be fully accurate. Self-selection bias may also be a factor, as parents who chose to participate in the survey might have stronger opinions about school meals compared to those who opted not to participate. Finally, data collection during the COVID-19 pandemic and the associated supply chain disruptions may have influenced parental perceptions.

### Recommendations for Policy and Practice

This study found a high prevalence of food insecurity among California parents, including those whose children are not eligible for FRPMs. This underscores the need for universal school meal policies to address child hunger more effectively than traditional eligibility-based programs. Food insecurity was also linked to higher participation in school breakfast, highlighting the importance of expanding access. Additionally, parents from food-insecure households reported more negative perceptions and stigma around school meals, emphasizing the need to eliminate stigma to boost participation and satisfaction by those most likely to benefit from universal school meals.

## 5. Conclusions

This study found that school meals served free of charge to all students during the pandemic were beneficial for households of all income levels in California. Meals were accessed at a higher rate by families reporting food insecurity, which was prevalent among families well beyond the federal eligibility limits for FRPMs. The study also revealed that parents in food-insecure households had poorer perceptions of school meals and reported more stigma than food-secure households. Therefore, to ease food insecurity and optimize the development of children and adolescents, it is important to continue facilitating access to school meals, improving their quality and appeal, addressing parental perceptions, and reducing stigma.

## Figures and Tables

**Table 1:** Characteristics of parents and their children attending a K-12 public or charter school in California, stratified by food security level.

Survey Respondent Characteristics	All (n = 1110)		Food Secure (n = 502)		Food Insecure (n = 608)		*p*-Value
	n	%	n	%	n	%	
Relationship with the Student							0.0001
Mother	954	85.9	411	81.9	543	89.3	
Father	112	10.1	72	14.3	40	6.6	
Other1	44	4.0	19	3.8	25	4.1	
Preferred Language							0.22
English	893	80.5	412	82.1	481	79.1	
Spanish	217	19.5	90	17.9	127	20.9	
Household Size							0.004
2–3 people	311	28.0	139	27.7	172	28.3	
4 people	373	33.6	193	38.5	180	29.6	
5 or more people	426	38.4	170	33.9	256	42.1	
Participant in SNAP/CalFresh ^1^ (yes)	474	42.7	172	34.3	302	49.7	0.0001
**Student Characteristics**	**n**	**%**	**n**	**%**	**n**	**%**	***p*** **-value**
School Level							0.06
Elementary school	651	58.6	314	62.6	337	55.4	
Middle school/Junior high	203	18.3	82	16.3	121	199	
High school	256	23.1	106	21.1	150	24.7	
Student’s Race/Ethnicity							0.01
Hispanic	574	51.7	244	48.6	330	54.3	
Non-Hispanic White	227	20.5	107	21.3	120	19.7	
Asian Asian/American	94	8.5	57	11.4	37	6.1	
Black/African American	93	8.4	45	9.0	48	7.9	
Non-Hispanic Other + Multiracial	122	11.0	49	9.8	73	12.0	
Student’s Gender							0.46
Female	453	40.8	204	40.6	249	41.0	
Male	449	40.5	211	42.0	238	39.1	
Other ^2^	208	18.7	87	17.3	121	19.9	
FRPM classification							0.0001
Free	484	43.6	147	29.3	337	55.4	
Reduced-Price	179	16.1	79	15.7	100	16.5	
Non-Eligible	447	40.3	276	55.0	171	28.1	
**Student Characteristics**	**Mean**	**SD^3^**	**Mean**	**SD**	**Mean**	**SD**	***p*** **-value**
Breakfast participation (days/week)	2.68	1.9	2.50	1.9	2.83	1.9	0.01
Lunch participation (days/week)	3.44	1.8	3.38	1.8	3.49	1.8	0.33

^1^ Supplemental Nutrition Assistance Program (SNAP), California’s food stamps program (CalFresh). ^2^ Other genders included nonbinary, transgender, ≥1 category, and preferred not to answer. ^3^ Standard deviation (SD).

**Table 2:** Perceptions about school breakfast and lunch of California parents of K-12 students stratified by household food security status.

	School Breakfast				School Lunch			
	All (n = 874)	Food Secure (n = 389)	Food Insecure (n = 485)	*p*-Value	All (n = 1110)	Food Secure (n = 480)	Food Insecure (n = 570)	*p*-Value
	%	% ^1^			%	% ^1^		
**Positive or neutral perceptions of school meals**								
My child usually likes the breakfasts/lunches served at school.	43.9	47.1	42.4	0.17	47.4	52.2	43.4	0.01
My child has enough time to eat breakfast/lunch at school.	47.2	50.8	45.2	0.10	54.2	61.4	47.9	0.0001
The school breakfast/lunch menu offers meals that are healthy.	46.1	51.8	41.8	0.003	44.0	48.7	39.2	0.002
The quality of the school breakfasts/lunches is good.	39.5	43.7	36.1	0.02	36.9	39.6	34.4	0.08
My child can get enough food at the school breakfast/lunch to get full.	44.3	51.4	38.9	0.0001	46.0	54.0	39.3	0.0001
My child thinks the school breakfasts/lunches taste good.	44.7	49.8	40.7	0.01	39.6	44.8	35.3	0.002
**Negative perceptions of school meals**								
My child gets tired of the same foods being served at school breakfast/lunch.	55.8	51.4	59.2	0.02	57.7	49.9	63.8	0.0001
I have concerns about the amount of sugar in school breakfasts/lunches.	35.2	36.3	34.6	0.61	34.3	32.8	36.2	0.24
My child prefers to eat breakfast at/bring food from home instead of eating the school breakfast/lunch.	49.0	48.8	49.5	0.83	48.7	46.0	50.6	0.15
I would prefer my child to eat breakfast at/bring food from home instead of eating the school breakfast/lunch.	37.5	35.1	39.3	0.21	36.7	35.7	36.9	0.69
I would prefer my child to have the option to eat school breakfast after the bell.	47.7	41.0	53.1	0.0001	-	-	-	-

^1^ The percentage of parents who agreed or strongly agreed with each perception was adjusted by race/ethnicity, urbanicity, and the total number of children living with the parent using logistic regression.

**Table 3:** Perceptions about school meals of California parents of K-12 students stratified by household food security status.

	All (n = 1050)	Food Secure (n = 480)	Food Insecure (n = 570)	*p*-Value
	%	% ^1^		
School meals can save my family money.	81.6	78.5	83.3	0.05
School meals can save my family time since we do not have to prepare breakfast and/or lunch for my child.	79.2	77.8	80.1	0.36
School meals can help to reduce stress for me/my family.	75.0	73.1	77.2	0.12
I think that eating school meals may benefit students academically.	57.5	57.1	58.4	0.65
I believe that school meals are only for children whose families have low incomes.	17.9	15.3	20.0	0.047
My child is (or would be) embarrassed to eat school meals.	11.7	8.3	15.0	0.001

^1^ The percentage of parents who agreed or strongly agreed with each perception was adjusted by race/ethnicity, urbanicity, and the total number of children living with the parent using logistic regression.

**Table 4:** Association between food insecurity and frequency of student participation in school meals, stratified by school level and free or reduced-price eligibility ^1^.

Stratification Variable	Sample	Breakfast Participation (n = 874)			Lunch Participation (n = 1050)		
		n	β	*p*-Value	n	β	*p*-Value
None	All	874	0.13	0.002	1050	0.03	0.35
School level	Elementary school	538	0.16	0.002	620	0.07	0.08
	Middle school	164	−0.003	0.98	192	0.008	0.92
	High school	172	0.21	0.06	238	−0.02	0.79
FRPM Eligibility	Free	391	0.05	0.43	457	−0.03	0.53
	Reduced-price	143	0.09	0.39	168	−0.06	0.51
	Non-eligible	340	0.19	0.01	425	0.11	0.04

^1^ Poisson regression models were adjusted by race/ethnicity, urbanicity, and the total number of children living with the parent.

## Data Availability

The datasets presented in this article are not readily available for privacy reasons. Requests to access the datasets should be directed to the corresponding author.

## Supplementary Materials

The following supporting information can be downloaded at https://www.mdpi.com/article/10.3390/nu16193375/s1, Table S1: Test for the interaction of food insecurity with school level and free and reduced-price meal eligibility.

## Appendix A

2023 Online Statewide Survey of Parents.
