# Supplementary material for: Household Food Insecurity Is Associated with Parental Perceptions of and Student Participation in School Meals
Source: Nutrients. 2024 Oct 4;16(19):3375. doi: 10.3390/nu16193375 (PMC11478978; doi:10.3390/nu16193375)
Supplement: Supplementary file 1 [file nutrients-16-03375-s001.zip › Appendix A - Parent Survey 2022.pdf]

Survey to be administered to parent/caregivers (including prior to in-depth interviews)

**Study:** [REDACTED]<br>**Purpose:** Document parents' perspectives about the opportunities and challenges presented by FSMFA to inform initial implementation, including completing 'school meal' application forms (formative evaluation)<br>**Target Dates:** Conducted April 2022<br>**Sample:** 1110 parents in CA will complete survey, 48 of which will also be interviewed<br>**Additional Data:** Brief survey will be completed before interview<br>**Conducted By:** [REDACTED]

THOUGHTS ABOUT SCHOOL MEALS

Thank you for agreeing to complete this survey about your and your child's opinions of school meals this year. This survey should take less than 20 minutes to complete. We'll combine all the results from this study and you, your child and your school district will not be identified with your responses. If you have any questions, please contact Principal Investigator, [REDACTED], at [REDACTED].

We are interested in your thoughts about the meals provided to students at your child's school (grades K–12), and how school meals may help your family. First, some general questions about your child.

If you have more than one child in school, **please think of your child who had the most recent birthday who is in grade K-12 when responding to these questions and answer the questions for that child**.

1. What grade is your child in?

K 1 2 3 4 5 6 7 8 9 10 11 12

2. What is the full name of your child's school? (Please write in your answer and do not use abbreviations) ____

3. What district is your child's school in? (Please write in your answer, if you know the name)

____

4. How has your child attended school this year?

◯ All in-person ◯ Hybrid (combination of in-person and remote) ◯ All remote

5. Does your child's school currently offer breakfast?

◯ Yes ◯ No ◯ Don't know

[If YES to breakfast in Q5]

6. In a typical week this school year, how often do you think your child eats a school breakfast (breakfast served by the school and NOT brought from home)?

◯ No days ◯ 1 day ◯ 2 days ◯ 3 days ◯ 4 days ◯ 5 days

7. In a typical week this school year, how often do you think your child eats a school lunch (lunch served by the school and NOT brought from home)?
◯ No days ◯ 1 day ◯ 2 days ◯ 3 days ◯ 4 days ◯ 5 days

8. Please think about a typical week before the Covid-19 pandemic (Fall of 2019). ***[For students in grades 2-12 ***SKIP FOR PARENTS WHO ANSWER GRADE K&1 in Q1]***
In a typical week before the pandemic when schools were completely in-person, how often do you think your child ate a school breakfast (breakfast served by the school and NOT brought from home)?
◯ No days ◯ 1 day ◯ 2 days ◯ 3 days ◯ 4 days ◯ 5 days

9. Continuing to think about pre-pandemic… ***[For students in grades 2-12 per Q1]***
In a typical week before the Covid-19 pandemic when schools were completely in-person (Fall of 2019), how often do you think your child ate a school lunch (lunch served by the school and NOT brought from home):
◯ No days ◯ 1 day ◯ 2 days ◯ 3 days ◯ 4 days ◯ 5 days

10. Now-think about the meals served at your child's school this year. Please ask your child for their thoughts if you are not sure. (As a reminder, if you have more than one child, please think of your child with the most recent birthday, who is in grade K-12).

SCHOOL BREAKFAST ***[SKIP LOGIC: Only if YES to breakfast in the school per Q5]***

11. Now think just about the school breakfast this school year. Even if your child does NOT eat breakfast at school, we would still like to learn more about your and your child's opinions of the school breakfast. How much do you agree with the following statements?
1=Strongly disagree 2=Disagree 3=Neither agree nor disagree 4=Agree 5=Strongly agree 6=Don't know

1) My child usually likes the breakfasts served at school.
2) My child has enough time to eat breakfast at school.
3) My child gets tired of the same foods being served at school breakfast.
4) The school breakfast menu offers meals that are healthy (nutritious and balanced).
5) I have concerns about the amount of sugar in school breakfasts.
6) The quality of the school breakfasts is good.
7) My child likes to eat the school breakfast to be with friends.
8) My child can get enough food at the school breakfast to get full.
9) My child prefers to eat breakfast at home or on the way to school, or not eat breakfast, instead of eating the school breakfast.
10) I would prefer my child to eat breakfast at home or on the way to school, or not eat breakfast, instead of eating the school breakfast.
11) I would prefer my child to have the option to eat school breakfast after the bell, during regular school hours (instead of before school begins).
12) My child thinks the school breakfasts taste good.

SCHOOL LUNCH

12. Now think about the school lunch this school year. Even if your child does NOT eat lunch at school, we would still like to learn more about your and your child's opinions of the school lunch. How much do you agree with the following statements?

1=Strongly disagree 2=Disagree 3=Neither agree nor disagree 4=Agree 5=Strongly agree 6=Don't know

1) My child usually likes the lunches served at school.
2) My child has enough time to eat lunch at school.
3) My child gets tired of the same foods being served at school lunch.
4) The school lunch menu offers meals that are healthy (nutritious and balanced).
5) I have concerns about the amount of sugar in school lunches.
6) The quality of the school lunches is good.
7) My child likes to eat the school lunch to be with friends.
8) My child can get enough food at the school lunch to get full.
9) My child prefers to bring food from home or buy food off campus (if it is available) instead of eating the school lunch
10) I would prefer my child to bring food from home or buy food off campus (if it is available) instead of eating the school lunch
11) My child chooses whether or not to eat the school lunch based on the menu that day.
12) My child thinks the school lunches taste good.

13. Now think about <u>school meals overall</u>. Even if your child does NOT eat breakfast or lunch at school, we would still like to learn more about your opinions. How much do you agree with the following statements?
1=Strongly disagree 2=Disagree 3=Neither agree nor disagree 4=Agree 5=Strongly agree 6=Don't know

1) School meals can save my family money.
2) School meals can save my family time since we do not have to prepare a breakfast and/or lunch for my child.
3) School meals can help to reduce stress for me/my family.
4) My child is (or would be) embarrassed to eat school meals.
5) I know what is being served in the cafeteria.
6) I believe that school meals are only for children whose families have low incomes.
7) I think that eating school meals may benefit students academically.

14. How do you prefer to get information from your child's school about the school meals? (mark all that apply)

Website
Email
Flier sent home with children
Phone
Social Media (such as Facebook, Twitter)
Text
Other (please specify): ______________________

<u>SCHOOL MEAL APPLICATIONS</u>

Next are a few questions about the school meal application process for free or reduced-price meals.

15. <u>Prior to the Covid-19 pandemic</u> (Fall of 2019), did you fill out school meal application or household income form for your child?
◯ Yes ◯ No ◯ Not sure

16. This school year, did you receive any information from your child's school about completing a school meal application (such as by email, phone call, mailed letter)?
◯ Yes ◯ No ◯ Not sure

[If Yes to Q16]
17. How did you receive information from your child/~~children~~'s school about the school meal application? (mark all that apply)
◯ Email ◯ Phone call ◯ Letter ◯ Social media ◯ Other (please specify): ____________

18. How do you prefer to get information from your child's school about the school meal application or household income form?
◯ Email ◯ Phone call ◯ Letter ◯ Social media ◯ Not applicable (I don't need to/want to fill out a school meal application) ◯ Other (please specify): ____________

19. Which of the following make it difficult or less likely that you will fill out a school meal application or household income form? (mark all that apply)
◯ I don't need to fill out an application
◯ I'm not sure if I need to fill out an application
◯ I don't have the time
◯ It is too difficult to fill out the application
◯ The applications are not in a language I use at home
◯ I do not want to share information about my family
◯ I need help filling out the application
◯ My child/children don't eat school meals often enough
◯ I don't understand why I am filling out the form. The school has not explained this to me.
◯ It is not difficult to fill out the application. I am likely to do it.
◯ Other (please specify): ________________________

[Skip if selected "I don't need to fill out an application" in Q19]
20. Would you be more likely to fill out a school meal application or a household income form if you thought there were additional benefits to schools?
◯ Yes
◯ No
◯ Don't know/Not sure

21. Do you think there are benefits to the school (outside the school meal program) if parents fill out a school meal application or a household income form?
◯ Yes: (Explain) ______________________________________
◯ No
◯ Don't know/Not sure

(If answered Yes to filling out an application in Q15)
22. Which of the following helped your decision to fill out a school meal application or a household income form this year or in the past? (mark all that apply)
◯ I received information from my school about filling out a school meal application.

◯ I needed to so that my child could have free or reduced-price school meals.
◯ I think there are other benefits to my child's school if I fill out a school meal application (such as more funding for the school).
◯ It was easy to fill out the application.
◯ The application was in a language I use at home.
◯ I knew the information that I shared about my family was confidential.
◯ Other (please specify): ___________________________.

[Skip if selected "I don't need to fill out an application" in Q20]
23. Which of the following make you more likely to fill out a school meal application or a household income form? (mark all that apply)
◯ If there were other benefits to the school (such as more funding for my child's school)
◯ If the school made it easier to fill out the application
◯ If the application could be completed online
◯ If the application were in a language I use at home
◯ If I knew the information I shared about my family was confidential
◯ If I had help to fill out the application
◯ If my kids ate school meals often enough
◯ If the form did not ask for a Social Security Number (SSN)
◯ Other (please specify): _________________________

ABOUT YOU

The next questions are about you, your child (with the most recent birthday in grade K-12), and your household.

24. How are you related to your child?
◯ Mother
◯ Father
◯ Grandparent
◯ Aunt/Uncle
◯ Legal guardian
◯ Other (please specify): _____________

25. What is your child's ethnicity? ◯ Hispanic/Latino ◯ NOT Hispanic/Latino

26. What is your child's race? (mark all that apply)
◯ Alaska Native/American Indian ◯ Asian/Asian American
◯ Black/African American ◯ Native Hawaiian/Other Pacific Islander
◯ White/Caucasian ◯ Other (please specify):
____

27. What is your child's gender? (mark all that apply)
◯ Girl ◯ Boy ◯ Non-binary ◯ Transgender ◯ Genderqueer
◯ Prefer to self-identify as: ________________ ◯ Prefer not to respond

28. How many total people are in your household (including adults and children)?
1 2 3 4 5 6 7 8 9+

29. How many total children under 18 years old live with you?
1 2 3 4 5 6 7 8 9+

30. How many total children in grades K-12 live with you?
1 2 3 4 5 6 7 8 9+

31. What was your family income before taxes in the past 12 months? It is ok to make your best guess.
[Responses based on number of people in household]

32. What is the highest level of education that *you* have completed?
◯ Some high school or less ◯ Completed high school ◯ Some college ◯ Completed college

These next questions are about the food eaten in your household in the last 12 months and whether you were able to afford the food you need.

Here are two statements that people have made about their food situation. For these statements, please mark whether the statement was often true, sometimes true, or never true for your household in the last 12 months (that is past year).
33. The food that we bought just didn't last, and we didn't have money to get more.
◯ Often true ◯ Sometimes true ◯ Never true ◯ Don't know

34. We couldn't afford to eat balanced meals.
◯ Often true ◯ Sometimes true ◯ Never true ◯ Don't know

35. In the last 12 months, did you or other adults in your household ever cut the size of your meals or skip meals because there wasn't enough money for food?
◯ Yes, almost every month ◯ Yes, some months but not every month ◯Yes, only 1 or 2 months ◯ No ◯ Don't know

36. In the last 12 months, did you ever eat less than you felt you should because there wasn't enough money for food?
◯ Yes ◯ No ◯ Don't know

37. In the last 12 months, were you every hungry but didn't eat because there wasn't enough money for food?
◯ Yes ◯ No ◯ Don't know

38. Do you currently participate in SNAP/CalFresh (also known as EBT, formerly called Food Stamps) to help pay for groceries?
◯ Yes ◯ No

39. Did your child receive the Pandemic Electronic Transfer Benefit or P-EBT card this school year (2021-2022)? (P-EBT is a card with money for buying groceries that families with school-aged children who qualify received in the mail)
◯ Yes ◯ No ◯ Don't know/Not sure

40. Would you like to receive a $20 electronic gift card in appreciation for taking this survey?

◯ Yes ◯ No

41. Would it be ok to contact you for a brief (online) interview after you complete this survey so that we can learn more about your opinions of school meals? You will receive a $50 gift card (and interviews can be scheduled at a day and time that you choose).

◯ Yes ◯ No

[if Yes to Q40 or 41]

42. Your email address: ______________________________________

We will only use this email address to contact you to schedule an interview or to email the gift card as a thank you for taking this survey.

43. Your phone number: ______________________________________

We will ONLY use this as a backup to contact you about scheduling an interview and/or for the gift card for your participation and for brief reminders before your scheduled interview. Your phone number will not be shared with anyone and will only be used for the reasons noted above. You can opt out of SMS text reminders at any time.

If you do not want us to send you SMS text reminders regarding your interview or as a backup to be in touch about the gift card, then do not enter a phone number.

44. Interviews can be done in English or Spanish. Which language would you like the interview to be?

◯ English ◯ Spanish

Thank you for sharing your input!
