# Supplementary material for: Household Food Insecurity Is Associated with Parental Perceptions of and Student Participation in School Meals
Source: Nutrients. 2024 Oct 4;16(19):3375. doi: 10.3390/nu16193375 (PMC11478978; doi:10.3390/nu16193375)
Supplement: Supplementary file 1 [file nutrients-16-03375-s001.zip › Supplementary Table S1.pdf]

Supplementary Table S1. Test for the interaction of food insecurity with school level and free and reduced-price meal eligibility in a study of perceptions of school meals among California parents of K-12 students.

Variable	Interaction Term[1]	*p*-value of the interaction term	
		**Breakfast**	**Lunch**
School Level	Food insecurity*Middle school	0.18	0.37
	Food insecurity*High school	0.82	0.32
FRPM Eligibility	Food insecurity*Reduced-price	0.95	0.55
	Food insecurity*Non-eligible	0.03	0.05

[1] Interaction terms were added to the Poisson regression model evaluating the association between food insecurity and student participation in school meals adjusting by race/ethnicity, urbanicity, and the total number of children under 18 years old that live with the parent (n = 874 for breakfast and n = 1,050 for lunch).
